# Dynamic Behavior of Positive Solutions for a Leslie Predator-Prey System with Mutual Interference and Feedback Controls

**DOI:** 10.1155/2014/637026

**Published:** 2014-01-20

**Authors:** Cong Zhang, Nan-jing Huang, Chuan-xian Deng

**Affiliations:** Department of Mathematics, Sichuan University, Chengdu, Sichuan 610064, China

## Abstract

We consider a Leslie predator-prey system with mutual interference and feedback controls. For general nonautonomous case, by using differential inequality theory and constructing a suitable Lyapunov functional, we obtain some sufficient conditions which guarantee the permanence and the global attractivity of the system. For the periodic case, we obtain some sufficient conditions which guarantee the existence, uniqueness, and stability of a positive periodic solution.

## 1. Introduction

Leslie [1] introduced the famous Leslie predator-prey system

(1) $$\begin{array}{c} \dot{x}\left(t\right)=x\left(t\right)\left[a-bx\left(t\right)\right]-P\left(x\right)y\left(t\right),\\ \dot{y}\left(t\right)=y\left(t\right)\left[e-f\frac{y\left(t\right)}{x\left(t\right)}\right], \end{array}$$

where *x*(*t*) and *y*(*t*) stand for the population (the density) of the prey and the predator at time *t*, respectively, and *P*(*x*) is the so-called predator functional response to prey.

In system (1), it has been assumed that the prey grows logistically with growth rate *a* and carries capacity *a*/*b* in the absence of predation. The predator consumes the prey according to the functional response *P*(*x*) and grows logistically with growth rate *e* and carrying capacity *x*/*f* proportional to the population size of the prey (or prey abundance). The parameter *f* is a measure of the food quality that the prey provides and converted to predator birth. Leslie introduced a predator-prey model, where the carrying capacity of the predator's environment is proportional to the number of prey, and still stressed the fact that there are upper limits to the rates of increasing of both prey *x* and predator *y*, which are not recognized in the Lotka-Volterra model. These upper limits can be approached under favorable conditions: for the predators, when the number of prey per predator is large; for the prey, when the number of predators (and perhaps the number of prey also) is small [2].

In population dynamics, the functional response refers to the numbers eaten per predator per unit time as a function of prey density. Kooij and Zegeling [3] and Sugie et al. [4] considered the following functional response:

(2) $$\begin{array}{c} P\left(x\right)=\frac{cx^{p}}{k+x^{p}}\;\left(k>0,c,p\;\;\text{are}\;\;\text{positive}\;\;\text{constants}\right). \end{array}$$

In fact, the functional response with *p* ≥ 1 has been suggested in [4].

It is easy to see that system (1) can be written as the following system:

(3) $$\begin{array}{c} \dot{x}\left(t\right)=x\left(t\right)\left[a-bx\left(t\right)\right]-\frac{cx^{p}\left(t\right)y\left(t\right)}{k+x^{p}},\\ \dot{y}\left(t\right)=y\left(t\right)\left[e-f\frac{y\left(t\right)}{x\left(t\right)}\right]. \end{array}$$

When *p* = 1 or *p* = 2 in system (3), the Leslie system (3) can be written as the following system:

(4) $$\begin{array}{c} \dot{x}\left(t\right)=x\left(t\right)\left[a-bx\left(t\right)\right]-\frac{cx\left(t\right)y\left(t\right)}{k+x},\\ \dot{y}\left(t\right)=y\left(t\right)\left[e-f\frac{y\left(t\right)}{x\left(t\right)}\right] \end{array}$$

or

(5) $$\begin{array}{c} \dot{x}\left(t\right)=x\left(t\right)\left[a-bx\left(t\right)\right]-\frac{cx^{2}\left(t\right)y\left(t\right)}{k+x^{2}\left(t\right)},\\ \dot{y}\left(t\right)=y\left(t\right)\left[e-f\frac{y\left(t\right)}{x\left(t\right)}\right], \end{array}$$

which were considered in [5].

The mutual interference between predators and prey was introduced by Hassell in 1971 [6]. During his research of the capturing behavior between hosts and parasites, he found that the hosts or parasites had the tendency to leave from each other when they met, which interfered with the hosts capturing effects. It is obvious that the mutual interference will be stronger while the size of the parasite becomes larger. Thus, Pan [7] studied a Leslie predator-prey system with mutual interference constant *m*:

(6) $$\begin{array}{c} \dot{x}\left(t\right)=x\left(t\right)\left[a\left(t\right)-b\left(t\right)x\left(t\right)\right]-a\left(t\right)x\left(t\right)y^{m}\left(t\right),\\ \dot{y}\left(t\right)=y\left(t\right)\left[d\left(t\right)-e\left(t\right)\frac{y\left(t\right)}{x\left(t\right)}\right], \end{array}$$

where *a*(*t*), *b*(*t*), *d*(*t*), *e*(*t*) ∈ *C*(*R*, *R*+) are bounded and periodic functions and *m* ∈ (0,1] is a constant. Some sufficient conditions are obtained for the permanence, attractivity, and existence of the positive periodic solution of the system (6).

On the other hand, ecosystems in the real world are continuously distributed by unpredictable forces which can result in changes in the biological parameters such as survival rates. Of practical interest in ecology is the question of whether or not an ecosystem can withstand those unpredictable disturbances which persist for a finite period of time. In the language of control variables, we call the disturbance functions as control variables, whereas, the control variables discussed in most literatures are constants or time dependent [8–10]. In recent, there are many works on the feedback controls (see, e.g., [11–19] and the references therein). Furthermore, in recent research on species, dynamics of the Leslie system has important significance; see [20–29] and the references therein for details.

Therefore, Chen and Cao [5] have investigated the following Leslie system with feedback controls:

(7) x˙1(t)=x1(t)[b(t)−a(t)x1(t)       −c(t)x1(t)x2(t)h2x22(t)+x12(t)−d(t)u1(t)],x˙2(t)=x2(t)[g(t)−f(t)x2(t)x1(t)−p(t)u2(t)],u˙1(t)=α1(t)−β1(t)u1(t)+γ1(t)x1(t),u˙2(t)=α2(t)−β2(t)u2(t)+γ2(t)x2(t),

where *x*_1_(*t*) and *x*_2_(*t*) denote the density of prey and predator at time *t*, respectively, *u*_*i*_(*t*)  (*i* = 1,2) are control variables, *b*, *e* ∈ *C*(*R*, *R*) are almost periodic functions of *t*, *a*, *c*, *d*, *f*, *p*, *α*_*i*_, *β*_*i*_, *γ*_*i*_ ∈ *C*(*R*, *R*_+_) (*i* = 1,2) are all nonnegative almost periodic functions of *t*, and *h* ^2^ is a positive constant denoting the constant of capturing half-saturation.

By using a comparison theorem and constructing a suitable Lyapunov function, they obtained some sufficient conditions for the existence of a unique almost periodic solution and the global attractivity of the solutions of the system (7). By using the comparison and continuation theorems, and based on the coincidence degree and by constructing a suitable Lyapunov function, Wang et al. [30] further obtained some sufficient and necessary conditions for the existence and global attractivity of periodic solutions of the system (7) with the periodic coefficients.

However, to the best of our knowledge, up to now, still no scholar has considered the general nonautonomous Leslie predator-prey system with mutual interference and feedback controls. In this paper, we will consider following the Leslie predator-prey system:

(8) x˙1(t)=x1(t)[b(t)−a(t)x1(t)        −c(t)x1p−1(t)x2m(t)k+x1p(t)−d(t)u1(t)],x˙2(t)=x2(t)[g(t)−f(t)x2(t)x1(t)−p(t)u2(t)],u˙1(t)=α1(t)−β1(t)u1(t)+γ1(t)x1(t),u˙2(t)=α2(t)−β2(t)u2(t)+γ2(t)x2(t),

where *x*_1_(*t*) and *x*_2_(*t*) denote the density of prey and predator at time *t*, respectively, *u*_*i*_(*t*) (*i* = 1,2) are control variables, *b*(*t*), *a*(*t*), *c*(*t*), *d*(*t*), *g*(*t*), *f*(*t*), *p*(*t*), *α*_*i*_(*t*), *β*_*i*_(*t*), *γ*_*i*_(*t*)  (*i* = 1,2) ∈ *C*(*R*, *R*_+_), *p* ≥ 1, *k* > 0 and *m* ∈ (0,1] are positive constants.

For the general nonautonomous case, by using differential inequality theory and constructing a suitable Lyapunov functional, we obtain some sufficient conditions which guarantee the permanence and the global attractivity of the system (8). For the periodic case, we obtain some sufficient conditions which guarantee the existence, uniqueness, and stability of a positive periodic solution of the system (8). We would like to mention that the conditions are related to the interference constant *m*.

## 2. Preliminaries

In this section, we state several definitions and lemmas which will be useful in proving the main results of this paper.

Let *R* and *R* ^*n*^, respectively, denote the set of all real numbers and the *n*-dimensional real Euclidean space, and *R*_+_^*n*^ denote the nonnegative cone of *R* ^*n*^. Let *f* be a continuous bounded function on *R* and we set *f* ^*u*^ = sup_*t*∈*R*_ *f*(*t*) and *f* ^*l*^ = inf_*t*∈*R*_ *f*(*t*).

Throughout this paper, we assume that the coefficients of the system (8) satisfy the following conditions:

(9) $$\begin{array}{c} \underset{i=1,2}{\min}\left\{a^{u},b^{u},c^{u},d^{u},f^{u},g^{u},p^{u},\alpha_{i}^{u},\beta_{i}^{u},\gamma_{i}^{u}\right\}>0,\\ \underset{i=1,2}{\max}\left\{a^{l},b^{l},c^{l},d^{l},f^{l},g^{l},p^{l},\alpha_{i}^{l},\beta_{i}^{l},\gamma_{i}^{l}\right\}<\infty . \end{array}$$

**Definition 1**

If ${\left(\bar{x}\left(t\right),\bar{y}\left(t\right),\bar{u}\left(t\right),\bar{v}\left(t\right)\right)}^{T}$ is a positive solution of the system (8) and (*x*(*t*),*y*(*t*),*u*(*t*),*v*(*t*))^*T*^ is any positive solution of system (8) satisfying

(10) limt→+∞|x¯(t)−x(t)|=0,  limt→+∞|y¯(t)−y(t)|=0,limt→+∞|u¯(t)−u(t)|=0,  limt→+∞|v¯(t)−v(t)|=0,

then we say that ${\left(\bar{x}\left(t\right),\bar{y}\left(t\right),\bar{u}\left(t\right),\bar{v}\left(t\right)\right)}^{T}$ is globally attractive.

**Lemma 2 (see [31])**

If *a* > 0, *b* > 0, and $\dot{x}\geq (\leq )\;\;b-ax$, when *t* ≥ *t*_0_ and *x*(*t*_0_) > 0, one has

(11) $$\begin{array}{c} x\left(t\right)\geq \left(\leq \right)\frac{b}{a}{\left[1+\left(\frac{ax\left(t_{0}\right)}{b}-1\right)e^{-a(t-t_{0})}\right]}^{-1}. \end{array}$$

**Lemma 3 (see [31])**

If *a* > 0, *b* > 0, and $\dot{x}\geq (\leq )\;x(b-ax)$, when *t* ≥ *t*_0_ and *x*(*t*_0_) > 0, one has

(12) $$\begin{array}{c} x\left(t\right)\geq \left(\leq \right)\frac{b}{a}{\left[1+\left(\frac{b}{ax\left(t_{0}\right)}-1\right)e^{-b(t-t_{0})}\right]}^{-1}. \end{array}$$

**Lemma 4 (see [32])**

If function *f* is nonnegative, integral, and uniformly continuous on [0, +*∞*), then

(13) $$\begin{array}{c} \underset{t\to +\infty }{\lim}f\left(t\right)=0. \end{array}$$

One assumes the following hypothesis holds:

(*H*_1_) *kb* ^*l*^ > *c* ^*u*^ *M*_1_^*p*−1^ *M*_2_^*m*^ + *kd* ^*u*^ *M*_3_;
(*H*_2_) *g* ^*l*^ > *p* ^*u*^ *M*_4_;
(*H*_3_) [*s*_2_ *f*(*t*)/*M*_1_ − *w*_2_ *γ*_2_(*t*) − *w*_1_ *γ*_2_(*t*) − *s*_1_ *mm*_2_^*m*−1^ *M*_1_^*p*−1^ *c*(*t*)/(*k* + *m*_1_^*p*^)]^*l*^ > 0;
(*H*_4_) [*w*_1_ *β*_1_(*t*)−*s*_1_ *d*(*t*)+*w*_2_ *β*_1_(*t*)]^*l*^ > 0;
(*H*_5_) [*w*_1_ *β*_2_(*t*)−*s*_2_ *p*(*t*)+*w*_2_ *β*_2_(*t*)]^*l*^ > 0;
(*H*_6_) for 1 ≤ *p* ≤ 2,
  (14) [s1a(t)−w1γ1(t)−w2γ1(t)+k(p−1)s1m2mM1p−2c(t)(k+M1p)2 −s1M12p−2c(t)(k+m1p)2−s2M2f(t)m12]l>0;
(*H*_7_)  for *p* > 2,
  (15) [s1a(t)−w1γ1(t)−w2γ1(t)+k(p−1)s1m2mm1p−2c(t)(k+M1p)2 −s1M12p−2c(t)(k+m1p)2−s2M2f(t)m12]l>0,
  where *s*_*i*_ and *w*_*i*_ (*i* = 1,2) are positive constants.

For any given the initial conditions of the system (8), *x*_*i*0_ = *x*_*i*_(*t*_0_) > 0, *u*_*i*0_ = *u*_*i*_(*t*_0_) > 0 (*i* = 1,2), it is not difficult to see that the corresponding solution (*x*_1_(*t*),*x*_2_(*t*),*u*_1_(*t*),*u*_2_(*t*))^*T*^ satisfies *x*_*i*_(*t*) > 0, *u*_*i*_(*t*) > 0 (*i* = 1,2), for *t* ≥ *t*_0_.

## 3. General Nonautonomous Case

In this section, we will explore the dynamics of the system (8) and present some results including the permanence and the global attractivity of the system.

**Theorem 5**

Suppose that system (8) satisfies the assumptions (*H*_1_) and (*H*_2_). Then system (8) is permanent; that is, any positive solution (*x*_1_(*t*),*x*_2_(*t*),*u*_1_(*t*),*u*_2_(*t*))^*T*^ of the system (8) satisfies

(16) $$\begin{array}{c} 0<m_{i}\leq \underset{t\to +\infty }{\lim\;\inf}x_{i}\left(t\right)\leq \underset{t\to +\infty }{\lim\;\sup}x_{i}\left(t\right)\leq M_{i},\\ 0<m_{2+i}\leq \underset{t\to +\infty }{\lim\;\inf}u_{i}\left(t\right)\leq \underset{t\to +\infty }{\lim\;\sup}u_{i}\left(t\right)\leq M_{2+i},\;i=1,2, \end{array}$$

where

(17) $$\begin{array}{c} M_{1}=\frac{b^{u}}{a^{l}},\;\;M_{2}=\frac{g^{u}b^{u}}{f^{l}a^{l}},\\ M_{3}=\frac{a^{l}\alpha_{1}^{u}+\gamma_{1}^{u}b^{u}}{a^{l}\beta_{1}^{l}},\;\;M_{4}=\frac{f^{l}a^{l}\alpha_{2}^{u}+\gamma_{2}^{u}g^{u}b^{u}}{f^{l}a^{l}\beta_{2}^{l}} \end{array}$$

with

(18) $$\begin{array}{c} m_{1}=\frac{kb^{l}-c^{u}M_{1}^{p-1}M_{2}^{m}-kd^{u}M_{3}}{ka^{u}},\\ m_{2}=\frac{m_{1}\left(g^{l}-p^{u}M_{4}\right)}{f^{u}},\\ m_{3}=\frac{\alpha_{1}^{l}+\gamma_{1}^{l}m_{1}}{\beta_{1}^{u}},\;\;m_{4}=\frac{\alpha_{2}^{l}+\gamma_{2}^{l}m_{2}}{\beta_{2}^{u}}. \end{array}$$

**Proof**

From the first equation of the system (8), it follows that

(19) $$\begin{array}{c} {\dot{x}}_{1}\left(t\right)\leq x_{1}\left(t\right)\left(b^{u}-a^{l}x_{1}\left(t\right)\right). \end{array}$$

Applying Lemma 3 to (19), for a small enough positive constant *ϵ* > 0, there exists a *T*_1_ > 0 enough large such that

(20) $$\begin{array}{c} x_{1}\left(t\right)\leq \frac{b^{u}}{a^{l}}+\epsilon =M_{1}+\epsilon ,\;\forall t>T_{1}. \end{array}$$

From the second equation of the system (8) and (20), it follows that, for *t* > *T*_1_,

(21) $$\begin{array}{c} {\dot{x}}_{2}\left(t\right)\leq x_{2}\left(t\right)\left[g^{u}-f^{l}\frac{x_{2}\left(t\right)}{\left(b^{u}/a^{l}+\epsilon \right)}\right]. \end{array}$$

Setting *ϵ* → 0 in inequality (21), we get

(22) $$\begin{array}{c} {\dot{x}}_{2}\left(t\right)\leq x_{2}\left(t\right)\left[g^{u}-f^{l}a^{l}\frac{x_{2}\left(t\right)}{b^{u}}\right]. \end{array}$$

For any *ϵ* > 0, it follows from (22) and Lemma 3 that there exists a *T*_2_ > *T*_1_ such that, for ant *t* > *T*_2_,

(23) $$\begin{array}{c} x_{2}\left(t\right)\leq \frac{g^{u}b^{u}}{f^{l}a^{l}}+\epsilon =M_{2}+\epsilon . \end{array}$$

By the third equation of system (8) and (20), we obtain

(24) $$\begin{array}{c} {\dot{u}}_{1}\left(t\right)\leq \alpha_{1}^{u}-\beta_{1}^{l}u_{1}\left(t\right)+\gamma_{1}^{u}\left(M_{1}+\epsilon \right). \end{array}$$

Letting *ϵ* → 0 in inequality (24), we get

(25) $$\begin{array}{c} {\dot{u}}_{1}\left(t\right)\leq \alpha_{1}^{u}+\gamma_{1}^{u}M_{1}-\beta_{1}^{l}u_{1}\left(t\right). \end{array}$$

For any *ϵ* > 0, it follows from (25) and Lemma 2 that there exists a *T*_3_ > *T*_2_ such that, for any *t* > *T*_3_,

(26) $$\begin{array}{c} u_{1}\left(t\right)\leq \frac{a^{l}\alpha_{1}^{u}+\gamma_{1}^{u}b^{u}}{a^{l}\beta_{1}^{l}}+\epsilon =M_{3}+\epsilon . \end{array}$$

By the forth equation of system (8) and (23), we have

(27) $$\begin{array}{c} {\dot{u}}_{2}\left(t\right)\leq \alpha_{2}^{u}-\beta_{2}^{l}u_{2}\left(t\right)+\gamma_{2}^{u}\left(M_{2}+\epsilon \right). \end{array}$$

Setting *ϵ* → 0 in inequality (27), we obtain

(28) $$\begin{array}{c} {\dot{u}}_{2}\left(t\right)\leq \alpha_{2}^{u}-\beta_{2}^{l}u_{2}\left(t\right)+\gamma_{2}^{u}M_{2}. \end{array}$$

For any *ϵ* > 0, it follows from (28) and Lemma 2 that there exists a *T*_4_ > *T*_3_ such that, for any *t* > *T*_4_,

(29) $$\begin{array}{c} u_{2}\left(t\right)\leq \frac{f^{l}a^{l}\alpha_{2}^{u}+\gamma_{2}^{u}g^{u}b^{u}}{\beta_{2}^{l}f^{l}a^{l}}+\epsilon =M_{4}+\epsilon . \end{array}$$

By the first equation of system (8), (20), (23), and (26), we obtain

(30) x˙1(t)≥x1(t)×[bl−aux1(t)−cu(M1+ϵ)p−1(M2+ϵ)mk  −du(M3+ϵ)].

Taking *ϵ* → 0 in inequality (30), we have

(31) $$\begin{array}{c} {\dot{x}}_{1}\left(t\right)\geq x_{1}\left(t\right)\left[b^{l}-\frac{c^{u}M_{1}^{p-1}M_{2}^{m}}{k}-d^{u}M_{3}-a^{u}x_{1}\left(t\right)\right]. \end{array}$$

In view of condition (*H*_1_) and Lemma 3, for any *ϵ* > 0, it follows from (28) that there exists a *T*_5_ > *T*_4_ such that, for any *t* > *T*_5_,

(32) $$\begin{array}{c} x_{1}\left(t\right)\geq \frac{kb^{l}-c^{u}M_{1}^{p-1}M_{2}^{m}-kd^{u}M_{3}}{ka^{u}}-\epsilon =m_{1}-\epsilon . \end{array}$$

From the second equation of the system (8), (29), and (32), we have

(33) $$\begin{array}{c} {\dot{x}}_{2}\left(t\right)\geq x_{2}\left(t\right)\left[g^{l}-f^{u}\frac{x_{2}\left(t\right)}{m_{1}-\epsilon }-p^{u}\left(M_{4}+\epsilon \right)\right]. \end{array}$$

Setting *ϵ* → 0 in inequality (33), we have

(34) $$\begin{array}{c} {\dot{x}}_{2}\left(t\right)\geq x_{2}\left(t\right)\left[g^{l}-p^{u}M_{4}-f^{u}\frac{x_{2}\left(t\right)}{m_{1}}\right]. \end{array}$$

In view of condition (*H*_2_) and Lemma 3, for any *ϵ* > 0, (34) shows that there exists a *T*_6_ > *T*_5_ such that, for any *t* > *T*_6_,

(35) $$\begin{array}{c} x_{2}\left(t\right)\geq \frac{m_{1}\left(g^{l}-p^{u}M_{4}\right)}{f^{u}}-\epsilon =m_{2}-\epsilon . \end{array}$$

By the third equation of system (8) and (32), we obtain

(36) $$\begin{array}{c} u_{1}\left(t\right)\geq \alpha_{1}^{l}-\beta_{1}^{u}u_{1}\left(t\right)+\gamma_{1}^{l}\left(m_{1}-\epsilon \right). \end{array}$$

Letting *ϵ* → 0 in inequality (36), we get

(37) $$\begin{array}{c} u_{1}\left(t\right)\geq \alpha_{1}^{l}+\gamma_{1}^{l}m_{1}-\beta_{1}^{u}u_{1}\left(t\right). \end{array}$$

For any *ϵ* > 0, it follows from (37) and Lemma 2 that there exists a *T*_7_ > *T*_6_ such that, for any *t* > *T*_7_,

(38) $$\begin{array}{c} u_{1}\left(t\right)\geq \frac{\alpha_{1}^{l}+\gamma_{1}^{l}m_{1}}{\beta_{1}^{u}}-\epsilon =m_{3}-\epsilon . \end{array}$$

By the forth equation of system (8) and (35), we have

(39) $$\begin{array}{c} u_{2}\left(t\right)\geq \alpha_{2}^{l}-\beta_{2}^{u}u_{2}\left(t\right)+\gamma_{2}^{l}\left(m_{2}-\epsilon \right). \end{array}$$

Taking *ϵ* → 0 in inequality (39), we get

(40) $$\begin{array}{c} u_{2}\left(t\right)\geq \alpha_{2}^{l}-\beta_{2}^{u}u_{2}\left(t\right)+\gamma_{2}^{l}m_{2}. \end{array}$$

For any *ϵ* > 0, by Lemma 2 and (40), there exists a *T*_8_ > *T*_7_ such that, for any *t* > *T*_8_,

(41) $$\begin{array}{c} u_{2}\left(t\right)\geq \frac{\alpha_{2}^{l}+\gamma_{2}^{l}m_{2}}{\beta_{2}^{u}}-\epsilon =m_{4}-\epsilon . \end{array}$$

Under the conditions (*H*_1_) and (*H*_2_), it follows from (20), (23), (26), (29), (32), (35), (38), and (41) that the system (8) is permanent. This completes the proof.

In the following, by constructing a suitable Lyapunov functional, we get the sufficient conditions for the globally attractive solution for the system (8).

**Theorem 6**

Suppose that system (8) satisfies the assumptions (*H*_1_)–(*H*_6_) or (*H*_1_)–(*H*_5_) and (*H*_7_). Then for any positive solution *z*(*t*) = (*x*_1_(*t*),*x*_2_(*t*),*u*_1_(*t*),*u*_2_(*t*))^*T*^ and $\bar{z}(t)={\left(y_{1}\left(t\right),y_{2}\left(t\right),v_{1}\left(t\right),v_{2}\left(t\right)\right)}^{T}$ of the system (8), one has

(42) $$\begin{array}{c} \underset{t\to +\infty }{\lim}\left|z\left(t\right)-\bar{z}\left(t\right)\right|=0. \end{array}$$

**Proof**

Let *z*(*t*) = (*x*_1_(*t*),*x*_2_(*t*),*u*_1_(*t*),*u*_2_(*t*))^*T*^ and $\bar{z}(t)={\left(y_{1}\left(t\right),y_{2}\left(t\right),v_{1}\left(t\right),v_{2}\left(t\right)\right)}^{T}$ be any positive solutions of the system (8). It follows from Theorem 5 that there exists *T*_9_ > 0 such that

(43) $$\begin{array}{c} m_{i}\leq x_{i}\left(t\right),\;\;y_{i}\left(t\right)\leq M_{i},\\ m_{2+i}\leq u_{i}\left(t\right),\;v_{i}\left(t\right)\leq M_{2+i}\;\left(i=1,2\right)\;\;\text{for}\;\;t>T_{9}. \end{array}$$

Consider the following functional:

(44) $$\begin{array}{c} V\left(t\right)=\sum_{i=1}^{2}\left[s_{i}\left|\ln x_{i}\left(t\right)-\ln y_{i}\left(t\right)\right|+w_{i}\sum_{i=1}^{2}\left|u_{i}\left(t\right)-v_{i}\left(t\right)\right|\right]. \end{array}$$

Calculating the upper right derivative of *V*(*t*) along the solution of the system (8), it follows that

(45) V+(t)=s1sgn(x1(t)−y1(t))[x˙1(t)x1(t)−y˙1(t)y1(t)] +w1sgn(u1(t)−v1(t))(u˙1(t)−v˙1(t)) +w1sgn(u2(t)−v2(t))(u˙2(t)−v˙2(t)) +s2sgn(x2(t)−y2(t))[x˙2(t)x2(t)−y˙2(t)y2(t)] +w2sgn(u1(t)−v1(t))(u˙1(t)−v˙1(t)) +w2sgn(u2(t)−v2(t))(u˙2(t)−v˙2(t))≤−s1a(t)|x1(t)−y1(t)|+s1d(t)|u1(t)−v1(t)| −w1β1(t)|u1(t)−v1(t)|+w1γ1(t)|x1(t)−y1(t)| −w1β2(t)|u2(t)−v2(t)|+w1γ2(t)|x2(t)−y2(t)| +s2p(t)|u2(t)−v2(t)|−w2β1(t)|u1(t)−v1(t)| +w2γ1(t)|x1(t)−y1(t)|−w2β2(t)|u2(t)−v2(t)| +w2γ2(t)|x2(t)−y2(t)|−s1c(t)sgn(x1(t)−y1(t)) ×[x1p−1(t)x2m(t)k+x1p(t)−y1p−1(t)y2m(t)k+y1p(t)] −s2f(t)sgn(x2(t)−y2(t))[x2(t)x1(t)−y2(t)y1(t)],  −s1c(t)sgn(x1(t)−y1(t))  ×[x1p−1(t)x2m(t)k+x1p(t)−y1p−1(t)y2m(t)k+y1p(t)] =−s1c(t)sgn(x1(t)−y1(t))  ×[x1p−1(t)x2m(t)k+x1p(t)−x1p−1(t)y2m(t)k+x1p(t)    +x1p−1(t)y2m(t)k+x1p(t)−y1p−1(t)y2m(t)k+y1p(t)] ≤s1c(t)x1p−1(t)k+x1p(t)|x2m(t)−y2m(t)|  −ks1c(t)y2m(t)(k+x1p(t))(k+y1p(t))|x1p−1(t)−y1p−1(t)|  +s1c(t)x1p−1(t)y1p−1(t)(k+x1p(t))(k+y1p(t))|x1(t)−y1(t)|.

By the mean value theorem and Theorem 5, in view of 0 < *m* ≤ 1, for *t* > *T*_9_, we have

(46) $$\begin{array}{c} \left|x_{2}^{m}\left(t\right)-y_{2}^{m}\left(t\right)\right|\leq mm_{2}^{m-1}\left|x_{2}\left(t\right)-y_{2}\left(t\right)\right|, \end{array}$$

(47) |x1p−1(t)−y1p−1(t)|≥(p−1)M1p−2|x1(t)−y1(t)|                  when  1≤p≤2,

(48) |x1p−1(t)−y1p−1(t)|≥(p−1)m1p−2|x1(t)−y1(t)|                    when  p>2.

Note that

(49) −s2f(t)sgn(x2(t)−y2(t))[x2(t)x1(t)−y2(t)y1(t)]  =−s2f(t)sgn(x2(t)−y2(t))   ×[x2(t)x1(t)−y2(t)x1(t)+y2(t)x1(t)−y2(t)y1(t)]  ≤−s2f(t)x1(t)|x2(t)−y2(t)|   +s2f(t)y2(t)x1(t)y1(t)|x1(t)−y1(t)|.

When 1 ≤ *p* ≤ 2, according to (45), (46), (47), and (49), it follows that, for *t* > *T*_9_,

(50) V+(t)≤−[s1a(t)−w1γ1(t)−w2γ1(t)   +k(p−1)s1M1p−2c(t)y2m(t)(k+x1p(t))(k+y1p(t))   −s1c(t)x1p−1(t)y1p−1(t)(k+x1p(t))(k+y1p(t))−s2f(t)y2(t)x1(t)y1(t)] ×|x1(t)−y1(t)| −[s2f(t)x1(t)−w2γ2(t)−w1γ2(t)−s1mm2m−1c(t)x1p−1(t)k+x1p(t)] ×|x2(t)−y2(t)| −[w1β1(t)−s1d(t)+w2β1(t)]|u1(t)−v1(t)| −[w1β2(t)−s2p(t)+w2β2(t)]|u2(t)−v2(t)|≤−[s1a(t)−w1γ1(t)−w2γ1(t)   +k(p−1)s1m2mM1p−2c(t)(k+M1p)2−s1M12p−2c(t)(k+m1p)2   −s2M2f(t)m12]|x1(t)−y1(t)| −[s2f(t)M1−w2γ2(t)−w1γ2(t)−s1mm2m−1M1p−1c(t)k+m1p] ×|x2(t)−y2(t)| −[w1β1(t)−s1d(t)+w2β1(t)]|u1(t)−v1(t)| −[w1β2(t)−s2p(t)+w2β2(t)]|u2(t)−v2(t)|.

When *p* > 2, according to (45), (46), (48), and (49), for *t* > *T*_9_, we have

(51) V+(t)≤−[s1a(t)−w1γ1(t)−w2γ1(t)   +k(p−1)s1m1p−2c(t)y2m(t)(k+x1p(t))(k+y1p(t))   −s1c(t)x1p−1(t)y1p−1(t)(k+x1p(t))(k+y1p(t))−s2f(t)y2(t)x1(t)y1(t)] ×|x1(t)−y1(t)| −[s2f(t)x1(t)−w2γ2(t)−w1γ2(t)−s1mm2m−1c(t)x1p−1(t)k+x1p(t)] ×|x2(t)−y2(t)| −[w1β1(t)−s1d(t)+w2β1(t)]|u1(t)−v1(t)| −[w1β2(t)−s2p(t)+w2β2(t)]|u2(t)−v2(t)|≤−[s1a(t)−w1γ1(t)−w2γ1(t)   +k(p−1)s1m2mm1p−2c(t)(k+M1p)2   −s1M12p−2c(t)(k+m1p)2−s2M2f(t)m12]|x1(t)−y1(t)| −[s2f(t)M1−w2γ2(t)−w1γ2(t)−s1mm2m−1M1p−1c(t)k+m1p] ×|x2(t)−y2(t)| −[w1β1(t)−s1d(t)+w2β1(t)]|u1(t)−v1(t)| −[w1β2(t)−s2p(t)+w2β2(t)]|u2(t)−v2(t)|.

When 1 ≤ *p* ≤ 2, it follows from (50) and assumptions (*H*_1_)–(*H*_6_) that there exist four positive constants *δ*_*j*_ (*j* = 1,2, 3,4) such that

(52) V+(t)≤−∑i=12δi|xi(t)−yi(t)|−∑i=12δi+2|ui(t)−vi(t)|,       ∀t>T9,

which shows that *V*(*t*) is nonincreasing on [*T*_9_, +*∞*). Integrating both sides of (52) on [*T*_9_, *t*), we get

(53) V(t)+∑i=12δi∫T9t|xi(t)−yi(t)|dt  +∑i=12δi+2∫T9t|ui(t)−vi(t)|dt≤V(T11)<+∞.

This implies that

(54) $$\begin{array}{c} \left|x_{i}\left(t\right)-y_{i}\left(t\right)\right|,\left|u_{i}\left(t\right)-v_{i}\left(t\right)\right|\in L^{1}\left(\left[T_{9},+\infty \right)\right),\;\;\;i=1,2. \end{array}$$

By Lemma 4, we have

(55) $$\begin{array}{c} \underset{t\to +\infty }{\lim}\left|x_{i}\left(t\right)-y_{i}\left(t\right)\right|=0,\;\;\underset{t\to +\infty }{\lim}\left|u_{i}\left(t\right)-v_{i}\left(t\right)\right|=0 \end{array}$$

for *i* = 1,2, and so

(56) $$\begin{array}{c} \underset{t\to +\infty }{\lim}\left|z\left(t\right)-\bar{z}\left(t\right)\right|=0. \end{array}$$

When *p* > 2, by similar way, from (51) and assumptions (*H*_1_)–(*H*_5_) and (*H*_7_), we can show that

(57) $$\begin{array}{c} \underset{t\to +\infty }{\lim}\left|z\left(t\right)-\bar{z}\left(t\right)\right|=0. \end{array}$$

This completes the proof.

## 4. Periodic Case

To this end, in order to get the existence and uniqueness of a positive periodic solution of the system (8), we further assume that system (8) satisfies the following condition.

(*H*_8_) *b*(*t*), *a*(*t*), *c*(*t*), *d*(*t*), *g*(*t*), *f*(*t*), *p*(*t*), *α*_*i*_(*t*), *β*_*i*_(*t*), *γ*_*i*_(*t*) ∈ *C*(*R*, *R*+), (*i* = 1,2) are all continuous, real-valued positive *ω*-periodic functions; *ω* > 0 is a constant.

Let *S* = {(*x*_1_(*t*), *x*_2_(*t*), *u*_1_(*t*), *u*_2_(*t*))^*T*^ ∈ *R*_+_^4^ | *m*_1_ ≤ *x*_1_(*t*) ≤ *M*_1_, *m*_2_ ≤ *x*_2_(*t*) ≤ *M*_2_, *m*_3_ ≤ *u*_1_(*t*) ≤ *M*_3_, *m*_4_ ≤ *u*_2_(*t*) ≤ *M*_4_}. Let *Z*(*t*, *Z*_0_) = (*x*_1_(*t*, *Z*_0_), *x*_2_(*t*, *Z*_0_), *u*_1_(*t*, *Z*_0_), *u*_2_(*t*, *Z*_0_))^*T*^.  for *t* ≥ *t*_0_ be the uniqueness solution of the system (8) with the initial condition *Z*_0_ = (*x*_1_(*t*_0_), *x*_2_(*t*_0_), *u*_1_(*t*_0_), *u*_2_(*t*_0_))^*T*^. By Theorem 5, there exists a *T*_0_ > 0 such that (*x*_1_(*t*), *x*_2_(*t*), *u*_1_(*t*), *u*_2_(*t*))^*T*^ ∈ *S*, for *t* ≥ *T*_0_.

Since *x*_1_(*t*, *Z*_0_) is continuous on [*t*_0_, *T*_0_], there exist two positive constants *m*_1_* and *M*_1_* such that *m*_1_* ≤ *x*_1_(*t*, *Z*_0_) ≤ *M*_1_* for *t* ∈ [*t*_0_, *T*_0_]. Let *l*_1_ = min{*m*_1_*, *m*_1_} and *L*_1_ = max{*M*_1_*, *M*_1_}. Then *l*_1_ ≤ *x*_1_(*t*, *Z*_0_) ≤ *L*_1_, for *t* > *t*_0_. Similarly, we can find positive constants *l*_2_ and *L*_2_ such that *l*_2_ ≤ *x*_2_(*t*, *Z*_0_) ≤ *L*_2_ and positive constants *l*_*i*_ and *L*_*i*_ such that *l*_*i*_ ≤ *u*_*i*−2_(*t*, *Z*_0_) ≤ *L*_*i*_ (*i* = 3,4) for *t* ≥ *t*_0_. Let S* = {(*x*_1_(*t*), *x*_2_(*t*), *u*_1_(*t*), *u*_2_(*t*))^*T*^ ∈ *R*_+_^4^ | *l*_1_ ≤ *x*_1_(*t*) ≤ *L*_1_, *l*_2_ ≤ *x*_2_(*t*) ≤ *L*_2_, *l*_3_ ≤ *u*_1_(*t*) ≤ *L*_3_, *l*_4_ ≤ *u*_2_(*t*) ≤ *L*_4_}. Therefore, we have *Z*(*t*, *Z*_0_) ∈ S*, for *t* ≥ *t*_0_. Define the Poincare mapping *A* : *R*_+_^4^ → *R*_+_^4^ as follows:

(58) $$\begin{array}{c} A\left(Z_{0}\right)=Z\left(\omega ,Z_{0}\right), \end{array}$$

where *ω* is defined in assumption (*H*_8_).

It is easy to see that the existence of periodic solution in (8) with assumptions (*H*_8_) is equal to prove that the mapping *A* has at least one fixed point.

**Theorem 7**

If the assumptions (*H*_1_), (*H*_2_), and (*H*_8_) hold, system (8) has at least one positive *ω*-periodic solution.

**Proof**

According to discussion above, we obtain *A*(S*) ⊂ S*. And we can get that the mapping *A* is continuous by the theory of ordinary differential equation. It is obvious that S* is a closed and convex set. Therefore, *A* has at least one fixed point by Brouwer fixed point theorem. That is to say, if system (8) satisfies (*H*_1_), (*H*_2_), and (*H*_8_), system (8) has at least one positive *ω*-periodic solution. This completes the proof.

Finally, some sufficient conditions are obtained for the uniqueness of the positive *ω*-periodic solution for the system (8).

**Theorem 8**

If system (8) satisfies (*H*_1_)–(*H*_6_) and (*H*_8_) or (*H*_1_)–(*H*_5_), (*H*_7_) and (*H*_8_), system (8) has only one positive *ω*-periodic solution which is globally attractive.

**Proof**

If system (8) satisfies (*H*_1_)–(*H*_6_) and (*H*_8_), (8) has at least one *ω*-periodic solution by Theorem 7. For any two positive *ω*-periodic solutions *z*(*t*) = (*x*_1_(*t*),*x*_2_(*t*),*u*_1_(*t*),*u*_2_(*t*))^*T*^ and $\bar{z}(t)={\left(y_{1}\left(t\right),y_{2}\left(t\right),v_{1}\left(t\right),v_{2}\left(t\right)\right)}^{T}$ of the system (8). We claim that $z(t)\equiv \bar{z}(t)$, for all *t* ∈ [0, *ω*]. Otherwise, there must be at least *ξ* ∈ [0, *ω*] such that *x*_1_(*ξ*) ≠ *y*_1_(*ξ*); that is, |*x*_1_(*ξ*) − *y*_1_(*ξ*)|∶ = *ρ* > 0. It follows that

(59) ρ=limn→+∞|x1(ξ+nω)−y1(ξ+nω)| =limt→+∞|x1(t)−y1(t)|>0,

which contracts with (42). Thus *x*_1_(*t*) ≡ *y*_1_(*t*), for all *t* ∈ [0, *ω*], hods. Similarly, we can prove that

(60) x2(t)≡y2(t),  u1(t)≡v1(t),  u2(t)≡v2(t),  ∀t∈[0,ω].

Therefore, the uniqueness of the periodic solution of the system (8) is obtained. By Theorem 6, if system (8) satisfies (*H*_1_)–(*H*_6_) and (*H*_8_), (8) has only one positive *ω*-periodic solution which is globally attractive. Similarly, we can prove that system (8) satisfying (*H*_1_)–(*H*_5_), (*H*_7_), and (*H*_8_) has a unique positive *ω*-periodic solution which is globally attractive. This completes the proof.
